# Research on the location model of emergency rescue facilities based on disaster risk—A Case study of earthquake disaster

**DOI:** 10.1371/journal.pone.0350148

**Published:** 2026-06-24

**Authors:** Qian Li, Xing Ju, Tuo Meng, Mengchu Xiao, Yi Xu, Tengjian Li, Xiaoqing Zhao

**Affiliations:** 1 ChinaAero Geophysical Survey&Remote Sensing Center for Natural Resources, Beijing, China; 2 National Research Center for Geoanalysis, CAGS, Beijing, China; 3 Service Bureau of the Ministry of Natural Resources, PRC, Beijing, China; 4 The Institute of Geology, Chinese Academy of Geological Sciences, Beijing, China; Mississippi State University, UNITED STATES OF AMERICA

## Abstract

Rational site selection for disaster relief supply reserve depots is crucial for mitigating natural disaster risks. This study constructs a site selection model for relief supply reserve depots based on an analysis of natural disaster risks. It identifies four disaster risk elements: hazard, exposure, vulnerability, and capacity by examining the definition of natural disaster risk and applying principles of disaster system theory. The relationships between these four elements and the selection of relief supply reserve depot locations are analyzed to develop the proposed site selection model. The model applies the entropy weight method and the geometric mean model to calculate comprehensive indicators across multiple regions, and the results for pre-disaster comprehensive regional factors are obtained. The model performs a targeted analysis of post-disaster regional losses by considering the relationships among hazard, exposure, vulnerability, and capacity and by distinguishing the risk characteristics of different disaster types. The study applies responsive technical methods to determine functional criteria for relief supply reserve depots using data sources such as regional GIS and satellite remote sensing data. Through multilevel constraint relationships, the model establishes a regional layout of multi-tiered relief supply reserve depots and ultimately integrates urban planning and other factors to determine candidate areas. The study is demonstrated through an extreme disaster scenario, specifically an earthquake of magnitude 7 or higher, in the western region of Yunnan, China. The resulting layout plan is relatively optimal, validating the effectiveness of the proposed model.

## 1. Introduction

The number of reported disaster events has increased annually over the past two decades based on statistics from the United Nations Office for Disaster Risk Reduction [1]. If the current trend continues, projections indicate that by 2030, the global annual occurrence of disasters could rise from approximately 400 events in 2015 to nearly 560 events. China is among the countries most severely affected by natural disasters. The government has consistently prioritized disaster prevention and mitigation initiatives and has established clear, quantifiable indicators to ensure the effectiveness of these efforts. Among these initiatives, establishing a comprehensive disaster relief supplies reserve system is of paramount importance. This system plays a critical role in safeguarding against widespread natural disasters and mitigating the impacts of extreme natural disasters [1]. As China enters the “14th Five-Year Plan” development phase [2], the government has been tasked with enhancing the layout of the emergency reserve system. Therefore, the current stage of research on site selection for disaster relief supplies reserve depots in China holds substantial practical application value.

The site selection for disaster relief supplies reserve depots is an essential component of natural disaster risk management. Several scholars [3–5] have comprehensively reviewed the site selection for disaster relief supplies reserve depots. Findings indicated that developing site selection models from interdisciplinary and multi-hazard integration perspectives to explore relief supplies reserve depot site selection problems under natural disaster scenarios has become a mainstream research trend. For instance, [6] developed a joint government-enterprise disaster relief supplies reserve depot location model in the context of rainstorms and large-scale geological disasters. They aimed to minimize the total cost and solved the model using the simulated annealing algorithm, considering victim deprivation costs, rescue timeliness, and transportation and construction costs. [7] constructed a multilevel coverage location model by addressing the temporal characteristics of earthquake supply distribution and depot capacity constraints. They utilized the maximum norm ideal point method to build a model that minimizes objective deviation across multiple goals, including maximizing rescue utility, balancing coverage quality, and ensuring timeliness equity. They compared the branch and bound algorithm with NSGA-II to solve it. [8] prioritized “differentiated response time” as a core indicator. They established a multi-factor comprehensive evaluation model for disaster relief supplies reserve depots to minimize total emergency costs and solve it using the Genetic Algorithm by integrating constraints such as transportation modes, vehicle types, and construction costs. [9] focused on the demand for supplies following strong earthquakes to develop a hierarchical disaster relief supplies reserve location model. They incorporated constraints including transportation efficiency satisfaction, depot quantity, and coverage area, solving the problem using a heuristic multi-center clustering algorithm to maximize coverage. [10] constructed a dynamic location model for livelihood supplies reserve depots in the context of severe floods. They proposed three solution schemes under dynamic demand scenarios in Zhejiang by incorporating the Pareto distribution principle and constraining the total transportation time and disaster loss. [11] utilized K-clustering of typhoon paths to weight disaster risk based on the linear distance from clustered paths to potential disaster zones. They constructed a hierarchical location model and validated it in Zhejiang by combining transportation efficiency, facility quantity, and distribution uniformity. [12] employed Fault Tree Analysis to determine disaster zone weights regarding earthquake rescue. With constraints on weights, transportation efficiency, and operating costs, they minimized costs and solved the model using particle swarm optimization combined with a local branching algorithm. Similarly, [13] integrated seismic belt distribution, construction costs, and rescue efficiency by focusing on earthquake scenarios. They developed a coverage maximization location model to obtain the optimal coverage scheme by introducing seismic risk weights and utilizing the DEA evaluation method. However, despite the abundance of research achievements, previous research has experienced a certain disconnect between the achievements in disaster studies and the current planning and emergency management practices of disaster relief supplies reserve systems. Two primary reasons were identified: First, prior studies on disaster risk analysis primarily focused on probabilistic factors while neglecting the intrinsic nature of disaster risk. This limitation reduces the accuracy of identifying hazardous areas, hinders the precise determination of service targets and constraints in site selection, and also leads to inadequate consideration of the disaster avoidance capability of disaster relief supplies reserve depots. Second, existing research has primarily focused on transportation costs and coverage areas, while overlooking the regional and functional requirements of emergency supplies reserve depots under extreme disaster conditions. Therefore, the candidate areas for site selection are often defined at an overly coarse scale, making it difficult to identify locations that fully meet the required standards and limiting the practical value of the selection results. In view of these limitations, this study analyzes the characteristics of natural disaster risk elements to develop a site selection model for disaster relief supplies reserve depots that incorporates natural disaster risks. This model enables the accurate identification of disaster-stricken areas and balances regional disaster risk with the disaster avoidance capability of the depots. Simultaneously, the study identifies constraints and configures algorithms to ensure the functional requirements of the disaster relief supplies reserve depots are fully considered by integrating site selection standards.

In previous research on site selection methods for disaster relief supplies reserve depots, scholars have frequently overlooked critical details when validating their selection models, particularly when using natural disaster cases. These oversights typically include neglecting factors such as the severity level of the natural disaster, the affected areas and populations, and the emergency response methods [14]. Such omissions can result in selection models that perform adequately for general or widespread natural disasters [1] but fail validation when extreme events, such as earthquakes of magnitude 7 or higher, typhoons of magnitude 16 or higher, or major floods, are considered [1]. Advances in interdisciplinary observation technologies, such as meteorology and geology, have made various natural disasters more predictable and manageable. This shift has enabled proactive risk prevention measures, earlier evacuation of affected populations, and the pre-positioning of disaster relief supplies. However, responding to extreme events, particularly earthquakes of magnitude 7 or higher, remains a significant challenge for emergency response efforts due to their infrequent occurrence, high destructiveness, and limited predictability [15]. Therefore, this study selects extreme natural disasters, specifically earthquakes with a magnitude of 7 or higher, as the empirical case for the site selection model to ensure that the constructed model can effectively address extreme natural disasters.

## 2. Methods

**Fig 1:**
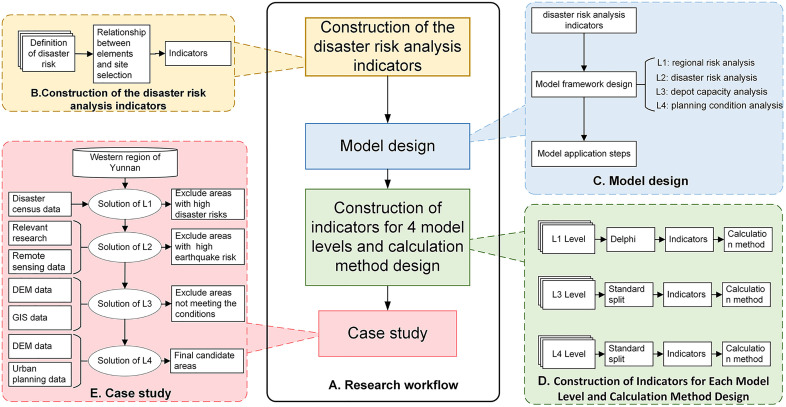
Research Workflow.

The workflow of this study is illustrated in Fig 1 based on the established research objectives. First, the study analyzes the association between natural disaster risk elements and site selection to develop an indicator system for natural disaster risk analysis. Then, a four-level site selection model is constructed based on this indicator system, and the application steps of the model are clarified. Building on this foundation, specific indicators for levels L1, L3, and L4 are designed using the Delphi method and the decomposition of site selection standards, and the corresponding calculation methods are determined. Finally, an empirical study is conducted using the western region of Yunnan Province as a case study. Following the model application steps, levels L1, L2, L3, and L4 are solved sequentially to determine the final candidate sites through a stepwise

### 2.1. The relationship of disaster risk elements to site selection

Current research primarily focuses on the HEV (Hazard–Exposure–Vulnerability) and HEVC (Hazard–Exposure–Vulnerability–Capacity) frameworks regarding the definition of natural disaster risk elements. Since these two frameworks differ in delineating risk components, inconsistent interpretations of the connotation of risk may arise, potentially leading to ambiguity in model construction and policy formulation. This study adopts the definition proposed by the United Nations International Strategy for Disaster Reduction (UNISDR) to ensure a unified understanding of natural disaster risk. UNISDR defines disaster risk as the potential casualties and property losses that a system, society, or community can suffer within a given period, determined probabilistically by the combined effects of hazard, exposure, vulnerability, and capacity [16]. The HEVC (Hazard–Exposure–Vulnerability–Capacity) framework is selected as the theoretical foundation for this study based on this definition. Three reasons support this choice. First, this definition is derived from the “Sendai Framework for Disaster Risk Reduction 2015–2030,” which was approved by the United Nations General Assembly in 2015, and enjoys broad international consensus [17]. Many signatory countries implemented risk-reduction efforts based on this framework. Second, the framework covers the period from 2015 to 2030, during which the understanding of disaster risk is unlikely to undergo significant changes, providing a stable theoretical foundation. Third, the definition includes the four elements of hazard, exposure, vulnerability, and capacity, which adequately fulfill the requirements for studying the site selection of disaster relief supplies reserve depots [18].

Based on this definition, this section conducts an in-depth analysis of the relationship between the respective elements and site selection, incorporating these factors into the site selection process. The core objective is to address limitations in the accuracy of identifying disaster-prone areas in existing studies, which makes it difficult to determine service targets and constraints for site selection. In this context, service targets refer to the population groups that the emergency supplies reserve depots are intended to serve, whereas constraints denote the conditions that must be satisfied during the site selection process. In addition, to identify locations that satisfy site selection standards, these standards must be integrated into the site selection model.

#### 2.1.1. Hazard analysis.

In site selection work, hazard analysis is reflected in two aspects. First, it assesses natural disaster hazards within a region where various types of natural disasters have already occurred and determines whether a higher-level disaster relief supplies reserve depot is required in that region (referred to as regional hazard element analysis, H1). Second, hazard analysis can determine the highest level of potential future disasters in regions with elevated natural disaster risks. It identifies areas exposed to disasters using risk assessment models that align with the characteristics of specific disasters, supporting exposure analysis (referred to as disaster hazard element analysis, H2). Through hazard analysis, hazardous areas can be delineated effectively during the site selection process.

#### 2.1.2. Exposure analysis.

Exposure analysis in site selection work is also twofold. First, it involves assessing exposure to disasters based on the analysis of natural disaster risks that the disaster relief supplies reserve depot needs to be particularly vigilant against. This analysis can support site selection efforts aimed at mitigating natural disaster risks (referred to as regional exposure element analysis, E1). Second, in regions with elevated natural disaster risks, exposure analysis can determine future affected areas and the number of affected people by applying techniques that match the disaster risk characteristics, supporting decisions regarding the scale of supply reserves and related aspects (referred to as disaster exposure element analysis, E2). Through exposure analysis, service targets can be determined effectively during the site selection process.

#### 2.1.3. Vulnerability analysis.

Vulnerability analysis in site selection also comprises two aspects. First, it assesses socio-economic vulnerability within a region, providing a comprehensive evaluation of urban development across different regions. This assessment can support decisions regarding the operational capacity of the reserve depots (referred to as regional vulnerability element analysis, V1). Second, vulnerability analysis focuses on the vulnerability of operational systems in disaster-prone areas. This information can assist decision-making related to material transportation methods, among other considerations (referred to as disaster vulnerability element analysis, V2).

#### 2.1.4. Capacity analysis.

Capacity analysis in site selection work has three aspects:

(1) It involves analyzing pre-disaster emergency response capacity within a region. This analysis can determine the overall emergency response capacity of the region, supporting site selection decisions associated with overall response capacity (referred to as regional capacity element analysis, C1).
(2) In regions with elevated natural disaster risks, capacity analysis can help calculate material transport capacity requirements aligned with emergency relief objectives. This approach can support the determination of operational capacity indicators for transportation equipment (referred to as disaster capacity element analysis, C2).
(3) Capacity analysis focuses on the capacity requirements of the reserve depots themselves. It can provide the primary conditions for selecting and constructing disaster relief supplies reserve depots (referred to as reserve depot capacity element analysis, C3).

### 2.2. Site selection model framework

#### 2.2.1. Model framework design.

The model establishes the post-disaster emergency functional objective of the site selection results as assisting disaster victims under specified conditions within a defined time frame. The model construction requirements are as follows:

Primary Considerations (regional risk analysis level, referred to as L1): Safety and reliability are the highest priorities. After clearly defining the regional reserve depot level, the analysis of the comprehensive environment of the site selection area should integrate areas exposed to natural disaster risks, regional operational capacity, and post-disaster emergency response capacity into the selection process.

Secondary Considerations (disaster risk analysis level, referred to as L2): Accessibility and extremeness are the main concerns at this level. The analysis focuses on a single type of high-risk natural disaster. It should determine the affected areas, the number of disaster victims, and the method of supplies transportation, and it should calculate the safe distance required for transportation equipment to deliver post-disaster emergency supplies.

Tertiary Considerations (reserve depot capacity analysis level, referred to as L3): At this level, the primary considerations are the functionality and timeliness of the disaster relief supplies reserve depot. The objective is to identify areas that meet the functional requirements of the reserve depot and to optimize transportation distance and time as the objective function.

Quaternary Considerations (planning condition analysis level, referred to as L4): This level mainly focuses on urban planning and other relevant factors, serving as a reference for determining the feasibility of solutions in the region where the optimal solution is located.

**Fig 2:**
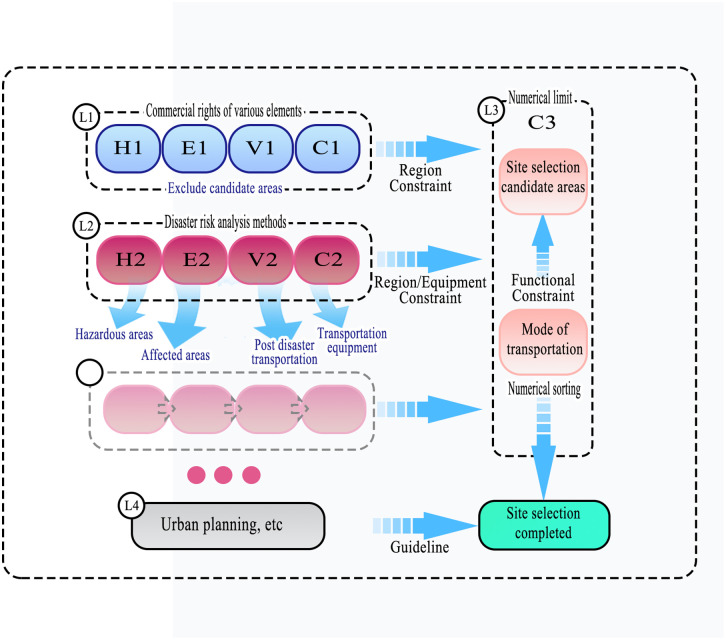
Basic framework of the site selection model based on natural disaster risk.

Based on these four aspects and considering the relationships among the various elements involved in site selection, the model is constructed, as depicted in Fig 2. The L1 and L2 levels in this model impose constraints on the L3 level. The L2 level increases progressively based on the accumulation of disaster types, resulting in multiple disaster-type sublevels labeled as L2_1_, L2_2_, and L2_3_.

#### 2.2.2. Steps for model application.

Based on the model framework shown in Fig 2, and to avoid situations in which overly strict constraints within the site selection model result in no feasible locations, the steps for applying the model are as follows:.

Step 1: Apply the L1 level by obtaining the analysis results of H1, E1, V1, and C1 within the region. Areas ranked lower in these analyses are designated as non-selectable zones. Data pertaining to L1 are sourced from the Statistical Yearbook of Yunnan Province for the most recent three years, as provided in the appendix.

Step 2: Apply the L2 level by analyzing H2, E2, V2, and C2 based on the specified disaster types, identifying the hazardous areas, service targets, transportation modes, and transportation equipment requirements necessary for depot site selection.

Step 3: Apply the L3 level by analyzing C3 in accordance with the requirements set by site selection standards to rank the candidate areas. Data related to L3 are sourced from the highway interchange location information provided in the appendix.

Step 4: Apply the L4 level by analyzing the areas of the top-ranked candidates based on urban planning requirements to determine their suitability for site selection. Data related to L4 are sourced from the information contained in the Statistical Yearbook of Yunnan Province and the municipal planning maps of Yunnan Province, as provided in the appendix.

Step 5: Select the emergency supplies reserve depots at each level based on the ranking of candidate areas.

### 2.3. Construction of indicators for each model level and calculation method design

In the basic framework shown in Fig 2, the L1 level involves a comprehensive analysis of potential locations, whereas the L2 level focuses on analyzing high-risk natural disasters. Given that different types of natural disasters have varying causative factors, the model construction process can only provide a generic design for the L1, L3, and L4 levels. The L2 level must be designed separately based on the specific characteristics of different disasters and the appropriate technical methods. Therefore, the primary focus of this research during the model construction phase is the development of models for the L1, L3, and L4 levels. This study uses China as an example, as it is a country with relatively favorable policies for constructing disaster relief supplies reserve depots. The research area includes three administrative levels: province, city, and county (district). The rules for locating disaster relief supplies reserve depots are as follows: if a provincial-level depot is required in a region, then the city and county (district) where the provincial-level depot is established will not set up other depot levels. Each city in the region requires one municipal-level depot; within that city, no depot will be established at the county level. Each county (district) in the region requires one county-level depot.

#### 2.3.1. Identification of the indicator and method design for L1.

Indicator identification: Selecting representative indicators to assess the extent of natural disaster losses is a practical approach for objectively revealing the overall losses and impacts caused by disasters in a given region [19]. Because constructing the L1 level involves a comprehensive analysis of the effects of natural disasters in the study area, this study employed the Delphi method. Three experts in natural disaster research, three experts in emergency management, and three experts in urban planning were invited to participate in a questionnaire survey. To ensure the reliability of data sources and the reproducibility of the research, the experts were asked to select indicators capable of reflecting the extent of natural disaster losses and the relevant requirements of site selection standards, based on the First National Comprehensive Risk Census Bulletin on Natural Disasters [20], the Yunnan Statistical Yearbook, and related research on site selection models for disaster relief supplies reserve depots. Through iterative consultations and feedback, the study eventually obtained evaluation indicators for the L1, L3, and L4 levels.

Method design: The evaluation indicators derived from expert opinions are multidimensional statistical quantities with different units; therefore, they must be normalized to facilitate further calculations. This study used the range transformation method [21] for data preprocessing. A higher indicator value in this method represents a higher degree or probability of disaster impact. The normalization formula is as follows:


$$\begin{array}{c} Y=\frac{X_{i}+\text{min}(X_{i})}{\text{max }\left(X_{i}\right)-\text{min}\left(X_{i}\right)} \end{array}$$ (1)

where *Y* is the normalized indicator; $X_{i}$ is the original value of the indicator; max($X_{i}$) and min($X_{i}$) are the maximum and minimum values of the indicator during the statistical period.

This study uses the entropy weight method to determine the weights of the indicators, ensuring objectivity and accuracy in the results. The geometric average model, which is not sensitive to datasets containing substantially larger individual indicators and is not prone to significant skewness in mean-biased distributions, is utilized to calculate the values of the indicators [22]. In this study, the steps for weighting indicators are as follows:

For *n* samples and *m* indicators, *x*_*i, j*_ are the values of the *j*-th indicator of the *i*-th sample (*i* = 1, 2,  ..., *n*; j = 1, 2, *m*).

The proportion of the *i*-th sample value in the *j-*th indicator can be described as follows:


$$\begin{array}{c} p_{i,j}=\frac{x_{i,j}}{\sum_{i=\text{1}}^{n}x_{i,j}},i=\text{1},...,n;j=\text{1},...,m \end{array}$$ (2)

The information entropy of the *j*-th indicator is defined as follows:


$$\begin{array}{c} e_{j}=-k\sum_{i=\text{1}}^{n}p_{i,j}ln(p_{i,j}),j=\text{1},...,m \end{array}$$ (3)

Where *k*=1/ln(*n*) >0, *e*_*j*_>0.

The redundancy (difference) of information entropy is calculated as follows:


$$\begin{array}{c} d_{j}=\text{1}-e_{j},j=\text{1},...,m \end{array}$$ (4)

The weights of each indicator are determined as follows:


$$\begin{array}{c} W_{j}=\frac{d_{j}}{\sum_{j=\text{1}}^{m}d_{j}},j=\text{1},...,m \end{array}$$ (5)

This study uses a geometric average model to calculate the comprehensive index to avoid data insensitivity caused by a single indicator being significantly too large. If 0 appears after normalization calculation, a small value of 0 is used for 01 replacement. The calculation formula is as follows:


$$\begin{array}{c} A_{i}=\sqrt[{t}]{{C_{i,\text{1}}W_{i,\text{1}}\times C_{i,\text{2}}W_{i,\text{2}}\times C_{i,\text{3}}W_{i,\text{3}}\times ...\times C}_{i,j}W_{i,j}} \end{array}$$ (6)

where *A* is the comprehensive absolute disaster index of a certain region; $C_{i,j}$ is the normalized value of the *j-*th specific indicator in the *i*-th sample; $W_{i,j}$ is the weight of the *j-*th specific indicator under the *i*-th sample, calculated by Equations (3)−(6); *t* is the total number of specific indicators.

#### 2.3.2. Indicators and calculation method for H1.

The primary function of a disaster relief supplies reserve depot is to respond to post-disaster emergencies. When assessing regional natural disaster risks, the magnitude of disaster losses can directly reflect the destructiveness of a disaster [23]. Therefore, when analyzing regional hazards, it is essential to use statistics on human and property losses to evaluate disaster severity. This assessment helps determine the disaster level, which, in turn, informs the required construction level of the reserve depot. Based on the typical reporting of disaster losses in existing post-disaster datasets, this study considers losses in terms of personnel, housing, and economic impacts, resulting in five specific C-level indicators (Table 1). These indicators are associated with nine types of natural disasters: drought, floods, earthquakes, geological hazards, typhoons, storms, low-temperature freezing disasters, snow disasters, and forest and grassland fires. Based on the modeling requirements of this study, if the study area includes more than three adjacent cities, these cities can be merged for mean calculations. If the disaster loss index in the combined area exceeds the mean value of these cities, a provincial-level reserve depot is deemed necessary. Based on this design, the formula for calculating the H1 indicator is extended from Equations (1) to (6) as follows:

**Table 1:** Evaluation index of regional natural disaster losses.

Level	Indicators		
A	Disaster damage assessment index ($\overset{\text{1}}{\underset{\text{1}}{\text{A}}}$)		
B	Personnel affected by disaster ($\overset{\text{1}}{\underset{\text{1}}{\text{B}}}$)	Building damage ($\overset{\text{1}}{\underset{\text{2}}{\text{B}}}$)	Economic loss ($\overset{\text{1}}{\underset{\text{3}}{\text{B}}}$)
C	Victims of disasters ($\overset{\text{1}}{\underset{\text{11}}{\text{C}}}$) Dead and missing persons ($\overset{\text{1}}{\underset{\text{12}}{\text{C}}}$)	Number of collapsed houses ($\overset{\text{1}}{\underset{\text{21}}{\text{C}}}$) Number of damaged houses ($\overset{\text{1}}{\underset{\text{22}}{\text{C}}}$)	Direct economic loss ($\overset{\text{1}}{\underset{\text{31}}{\text{C}}}$)


$$\begin{array}{cc} & A_{s}=Q_{x}A_{x}+Q_{y}A_{y}+...+{Q_{z}A}_{z}=Q_{x}\sqrt[{t}]{{C_{i,\text{1}}W_{i,\text{1}}\times C_{i,\text{2}}W_{i,\text{2}}\times C_{i,\text{3}}W_{i,\text{3}}\times ...\times C}_{i,j}W_{i,j}}\\ & +Q_{y}\sqrt[{t}]{{C_{i,\text{1}}W_{i,\text{1}}\times C_{i,\text{2}}W_{i,\text{2}}\times C_{i,\text{3}}W_{i,\text{3}}\times ...\times C}_{i,j}W_{i,j}}+...+Q_{z}\sqrt[{t}]{{C_{i,\text{1}}W_{i,\text{1}}\times C_{i,\text{2}}W_{i,\text{2}}\times C_{i,\text{3}}W_{i,\text{3}}\times ...\times C}_{i,j}W_{i,j}} \end{array}$$ (7)

where $A_{s}$ is the total comprehensive absolute disaster index of multiple regions; $A_{x}$, $A_{y}$,  ..., and $A_{z}$ are the sub-regions; $Q_{x},Q_{y}$,  ..., and $Q_{z}$ are the weighted values of the disaster index for each sub-region.

#### 2.3.3. Indicators and calculation method for E1.

Disaster relief supplies reserve depots are typical storage facilities, and geohazards and other surrounding site conditions can be managed during construction. Therefore, when analyzing exposure, it is essential to focus on the types of natural disasters that disaster relief supplies reserve depots are expected to mitigate. In this context, the analysis emphasizes water-related disasters, such as floods, typhoons, and heavy rainfall [24], reflecting the safety dimension of the model. Based on established post-disaster data collection methods, the indicators utilized to assess the impact of water-related disasters on disaster relief supplies reserve depots include disaster severity and economic losses, resulting in four specific C-level indicators (Table 2). Based on the modeling requirements of this study, the E1 indicator is calculated for all counties. When selecting a location for a provincial-level reserve depot, counties ranked in the top 15% for disaster severity within the study area are excluded. The formula for calculating this indicator is extended from Equations (1) to (7) as follows:

**Table 2:** Regional flood severity assessment indicators.

Level	Indicators	
A	Disaster assessment index ($\overset{\text{2}}{\underset{\text{1}}{\text{A}}}$)	
B	Disaster area ($\overset{\text{2}}{\underset{\text{1}}{\text{B}}}$)	Economic loss ($\overset{\text{2}}{\underset{\text{2}}{\text{B}}}$)
C	Annual frequency of disasters ($\overset{\text{2}}{\underset{\text{11}}{\text{C}}}$) Flood level condition ($\overset{\text{2}}{\underset{\text{12}}{C}}$) Waterlogged area ($\overset{\text{2}}{\underset{\text{13}}{\text{C}}}$)	Direct economic loss ($\overset{\text{2}}{\underset{\text{21}}{\text{C}}}$)


$$\begin{array}{c} D_{\text{p}-\text{c}}=\{A_{\text{1}},A_{\text{2}},A_{\text{3}},...,A_{p},...,A_{P}\} \end{array}$$ (8)

$$\begin{array}{c} \text{sort}{(D}_{\text{p}-\text{c}})=\{\begin{array}{c} \text{Exclusion}\\ \overbrace{A^{\text{1}},A^{\text{2}},A^{\text{3}}} \end{array}\begin{array}{c} \text{Reservation}\\ \overbrace{,...,A^{q},...,A^{P}} \end{array}\} \end{array}$$ (9)

where $D_{\text{p}-\text{c}}$ is the collection of county-level units within the provincial unit; *P* is the total number of county-level units within the region; $A_{p}$ is the *p*-th county-level unit, $\text{sort}{(D}_{\text{p}-\text{c}})$ is the ordering of elements in the collection from small to large; $A^{q}$ is the *q*-th county-level unit after the ordering.

When selecting the site for the municipal depot, the county-level unit with the highest disaster index ranking within each municipal unit is excluded, and the calculation formula is extended based on Equations (1)–(7). Accordingly, the formula is:


$$\begin{array}{c} D_{\text{m}-\text{c}}=\{A_{\text{1}},A_{\text{2}},A_{\text{3}},...,A_{p},...,A_{P}\} \end{array}$$ (10)

$$\begin{array}{c} \text{sort}{(D}_{\text{m}-\text{c}})=\{\begin{array}{c} \text{Exclusion}\\ \overbrace{A^{\text{1}}} \end{array}\begin{array}{c} \text{Reservation}\\ \overbrace{{,A^{\text{2}},A^{\text{3}},...,A}^{q},...,A^{P}} \end{array}\} \end{array}$$ (11)

where $D_{\text{m}-\text{c}}$ is the collection of county-level units within the municipal unit.

#### 2.3.4. Indicators and calculation method for V1.

The effective operation of a large-scale disaster relief supplies reserve depot requires a high level of comprehensive urban management capacity. Therefore, when selecting assessment indicators, it is necessary to evaluate essential service provision capacity, the quality of urban infrastructure, and environmental conditions. This evaluation yields nine specific C-level indicators (Table 3), representing the reliability dimension of the model. Based on the modeling requirements of this study, the V1 indicator is calculated for all counties. When selecting locations for provincial-level or municipal-level reserve depots, the county with the highest urban management capacity index within the study area is excluded. Based on this design, the formula for calculating the V1 indicator is extended from Equations (2)–(7),(10),(12).

**Table 3:** Indicators for operational capacity assessment.

Level	Indicators		
A	Operational capability assessment index ($\overset{\text{3}}{\underset{\text{1}}{\text{A}}}$)		
B	Basic living security ($\overset{\text{3}}{\underset{\text{1}}{\text{B}}}$)	Urban infrastructure ($\overset{\text{3}}{\underset{\text{2}}{\text{B}}}$)	Environmental level ($\overset{\text{3}}{\underset{\text{3}}{\text{B}}}$)
C	Water penetration rate ($\overset{\text{3}}{\underset{\text{11}}{\text{C}}}$) Gas penetration rate ($\overset{\text{3}}{\underset{\text{12}}{\text{C}}}$)	Density of water supply pipeline in built-up area ($\overset{\text{3}}{\underset{\text{21}}{\text{C}}}$) Road network density in built-up area ($\overset{\text{3}}{\underset{\text{22}}{\text{C}}}$) Drainage pipe density in built-up area ($\overset{\text{3}}{\underset{\text{23}}{\text{C}}}$) Sewage treatment rate ($\overset{\text{3}}{\underset{\text{24}}{\text{C}}}$)	Per capita green park area ($\overset{\text{3}}{\underset{\text{31}}{\text{C}}}$) Green coverage rate of built-up area ($\overset{\text{3}}{\underset{\text{32}}{\text{C}}}$) Domestic waste disposal rate ($\overset{\text{3}}{\underset{\text{33}}{\text{C}}}$)


$$\begin{array}{c} \text{sort}{(D}_{\text{m}-\text{c}})=\{\begin{array}{c} \text{Exclusion}\\ \overbrace{A^{\text{1}},...{,A}^{P-\text{1}}} \end{array}\begin{array}{c} \text{Reservation}\\ \overbrace{A^{P}} \end{array}\} \end{array}$$ (12)

#### 2.3.5. Indicators and calculation method for C1.

Large-scale disaster relief supplies reserve depots require strong emergency support to effectively implement post-disaster emergency response actions. These depots must demonstrate robust post-disaster emergency response capabilities to fulfill their operational roles after a disaster. Therefore, indicator selection for establishing disaster relief supplies reserve depots involves evaluating capacity in essential infrastructure support, rescue operations, and monitoring capability. This evaluation results in five specific C-level indicators (Table 4), further demonstrating the reliability of the model framework. In accordance with the modeling requirements in this study, the C1 indicator is calculated for all counties. When determining locations for provincial-level or municipal-level depots, county-level regions ranked lowest in operational capability indicators are excluded. Based on this design, the calculation formula for the C1 indicator includes Equations (2)–(7),(10),(12).

**Table 4:** Indicators of emergency capacity assessment.

Level	Indicators		
A	Emergency response capacity assessment Index ($\overset{\text{4}}{\underset{\text{1}}{\text{A}}}$)		
B	Infrastructure support ($\overset{\text{4}}{\underset{\text{1}}{\text{B}}}$)	Rescue support ($\overset{\text{4}}{\underset{\text{2}}{\text{B}}}$)	Monitoring support ($\overset{\text{4}}{\underset{\text{3}}{\text{B}}}$)
C	Communication base station density ($\overset{\text{4}}{\underset{\text{11}}{\text{C}}}$) Proportion of 10,000 emergency communication vehicles ($\overset{\text{4}}{\underset{\text{12}}{\text{C}}}$) Proportion of hospitalized beds per 10,000 people ($\overset{\text{4}}{\underset{\text{13}}{\text{C}}}$) Proportion of ambulances per 10,000 people ($\overset{\text{4}}{\underset{\text{14}}{\text{C}}}$) Proportion of investment in disaster prevention and mitigation ($\overset{\text{4}}{\underset{\text{15}}{\text{C}}}$)	Proportion of firefighters per 10,000 people ($\overset{\text{4}}{\underset{\text{21}}{\text{C}}}$) Proportion of fire engines per 10,000 people ($\overset{\text{4}}{\underset{\text{22}}{\text{C}}}$) Proportion of earthquake rescue workers per 10,000 people ($\overset{\text{4}}{\underset{\text{23}}{\text{C}}}$) Proportion of maritime rescue workers per 10,000 people ($\overset{\text{4}}{\underset{\text{24}}{\text{C}}}$) Proportion of rescue helicopters per 10,000 people ($\overset{\text{4}}{\underset{\text{25}}{\text{C}}}$)	Density of meteorological station ($\overset{\text{4}}{\underset{\text{31}}{\text{C}}}$) Density of hydrological station ($\overset{\text{4}}{\underset{\text{32}}{\text{C}}}$) Network density of seismic stations ($\overset{\text{4}}{\underset{\text{33}}{\text{C}}}$) Density of ground disaster monitoring sites ($\overset{\text{4}}{\underset{\text{34}}{\text{C}}}$) Density of Marine disaster monitoring sites ($\overset{\text{4}}{\underset{\text{35}}{\text{C}}}$)

#### 2.3.6. Indicators and technical methods for L3.

Within the L3 framework, there is only one element, C3, which focuses on assessing the functionality of disaster relief supplies reserve depots. Based on the requirements of the Standards for the Construction of Disaster Relief Supplies Reserve Depots, issued by the Ministry of Civil Affairs of the People’s Republic of China in 2009 [25], this study categorizes the functionality of disaster relief supplies reserve depots and establishes specific indicators. Details of these indicators are listed in Table 5 and 6. The final candidate areas that satisfy the three functional criteria for disaster relief supplies reserve depots are determined using regional GIS data to obtain road network information, identify transportation hubs, and determine candidate areas, and utilizing DEM (Digital Elevation Model) data to identify suitable flat and open terrain. The formula for determining the candidate area is specified in [26]:

**Table 5:** Construction standard site selection conditions decomposition and quantitative basis.

Number	Construction standard requirements	Function	Index	Technical means
Premise	Comply with local urban planning and follow the principles of safe storage and convenient transport	Convenient transportation	The transportation hub point should be selected as the intersection point of two highways	Identify transportation hubs based on road network data
1	High terrain, favorable engineering, and hydrogeological conditions	--	--	--
		--	--	--
2	Favorable municipal conditions	--	--	--
3	Kept away from fire sources and inflammable or explosive factories and warehouses	--	--	--
4	Convenient traffic and transport conditions, adjacency to a railway freight station or highway entrance in the case of municipal or higher-level relief supply warehouses	Adjacent to a highway entrance or a railway freight station	The candidate area is within 5 km of the entrance of a highway or railway freight station [27,28]	Regional GIS data identification to identify candidate areas
		Convenient transportation		
5	Flat terrain, broad vision, convenient for the emergency take-off and landing of helicopters in the case of municipal or higher-level relief supply warehouses	The terrain is flat, and the view is relatively wide	With the 150 × 150 m window as the unit, the slope of the unit involved in the candidate area must have less than 25% of the unit [27,28]	DEM data identifies flat open areas
		Helicopter take-off and landing		

**Table 6:** Scales of relief supply warehouses (Ministry of Civil Affairs of the People’s Republic of China, 2009).

Scale classification		Number of persons re-accommodated in an emergency (10,000)	Total building area (m^2^)	Warehouse (m^2^)
Central level (Regional)	Large	72−86	21800−25700	19563−23368
	Medium	54−65	16700−19800	14673−17661
	Small	36−43	11500−13500	9781−11684
Provincial level		12−20	5000−7800	3985−6641
Municipal level		4−6	2900−4100	2213−3321
County level		0.5−0.7	630−800	394−552


$$\begin{array}{c} a=\text{6378137m} \end{array}$$ (13)

$$\begin{array}{c} b=\text{6356752m} \end{array}$$ (14)

$$\begin{array}{c} e=\sqrt{{\text{1}-\left(\frac{b}{a}\right)}^{\text{2}}} \end{array}$$ (15)

$$\begin{array}{c} \text{e}\text{'}=\sqrt{{\left(\frac{a}{b}\right)}^{\text{2}}-\text{1}} \end{array}$$ (16)

$$\begin{array}{c} K=N_{B_{\text{0}}}*\text{cos}B_{\text{0}}=\frac{\frac{a^{\text{2}}}{b}}{\sqrt{\text{1}+e^{\prime \text{2}}*{(\text{cos}B_{\text{0}})}^{\text{2}}}}*\text{cos}B_{\text{0}} \end{array}$$ (17)

$$\begin{array}{c} x=K\text{ln}\left[\text{tan}(\frac{\pi }{\text{4}}+\frac{B}{\text{2}})*{(\frac{\text{1}-e\text{sin}B}{\text{1}+e\text{sin}B})}^{\frac{e}{\text{2}}}\right] \end{array}$$ (18)

$$\begin{array}{c} y=K(L-L_{\text{0}}) \end{array}$$ (19)

where *L* is longitude; *B* is latitude; *L*_*0*_ is the standard longitude (self-defined); *B*_*0*_ is the standard latitude (self-defined); (*x, y*) is the converted coordinates; *a* denotes the semi-major axis of the ellipsoid; *b* is the semi-minor axis of the ellipsoid; *e* denotes the first eccentricity; *e’* is the second eccentricity; $N_{B_{\text{0}}}$ is the radius of curvature in prime vertical; *K* is the radius of the parallel circle at the longitude and latitude position (*L*_*0,*_ *B*_*0*_).

After the DEM data is obtained, the calculation formula for identifying each selection is as follows:

Elevation mean value computation formula:


$$\begin{array}{c} \mu =\frac{\sum_{i=\text{1}}^{N}Xi}{N} \end{array}$$ (20)

Elevation variance calculation formula:


$$\begin{array}{c} \sigma^{\text{2}}=\frac{\sum_{i=\text{1}}^{N}{\left(Xi-\mu \right)}^{\text{2}}}{N} \end{array}$$ (21)

Elevation standard deviation calculation formula:


$$\begin{array}{c} \sigma =\sqrt{\sigma^{\text{2}}}=\sqrt{\frac{\sum_{i=\text{1}}^{N}{\left(Xi-\mu \right)}^{\text{2}}}{N}} \end{array}$$ (22)

where Xi is the elevation of each pixel; N is the number of regional pixels.

Based on the Notice of the State Council on the Issuance of the “14th Five-Year Plan” for the National Emergency Response System [2], affected individuals are required to receive effective assistance for basic living needs within 10 hours after the occurrence of a disaster or accident. Based on the model construction requirements, the first batch of essential relief supplies to a provincial-level disaster relief supplies reserve depot is required to be delivered within 5 hours.

#### 2.3.7. Indicators and technical methods for L4.

The L4 level serves as a reference for selecting locations for disaster relief supplies reserve depots. Its primary objective is to ensure that the selected areas are consistent with urban planning requirements. When conflicts arise between these objectives, decision-makers must evaluate reserve depot site selection within the broader context of urban planning constraints. In accordance with the Construction Standards for Disaster Relief Supplies Reserve Depots [25], L4 primarily applies to the construction of reserve depots above the municipal level. Therefore, L4 considers key planning indicators, including airspace restrictions, mineral resource zones, and water resource protection areas. These constraints can be identified using regional GIS data.

### 2.4. Case study

Western Yunnan Province, China, is located within the Yunnan-Burma massif along the southeastern margin of the Qinghai-Tibet Plateau. Historical seismic activity in China provides evidence that this region can generate earthquakes with magnitudes exceeding 7.0. These events include the 7.0 magnitude Gengma earthquake in 1941, the 7.3/7.4 magnitude Mangshi-Longling earthquake in 1976, and the 7.2 magnitude Mong Hpayak District earthquake that occurred within Myanmar’s borders in 2011 [5,29]. These events indicate that the area is highly seismically active and can remain a high-risk zone for future earthquakes exceeding magnitude 7.0. Therefore, this research selected the western part of Yunnan Province, China, as the study area (located between 23° N−26° N and 97° E−101° E). Data on natural disasters and urban development in the research area were obtained from the First National Comprehensive Risk Census Bulletin on Natural Disasters and the 2024 Yunnan Statistical Yearbook.

## 3. Results of a case study

### 3.1. The solution of L1

This study sets the data collection period for elements H1 and E1 from 1978 to 2020 by reviewing historical statistical data for Yunnan Province and ensuring that the statistical records accurately represent regional conditions. The data collection period for elements V1 and C1 is set to 2019 to avoid potential impacts associated with COVID-19 prevention and control measures. The research area covers five cities in Yunnan Province: Dali, Baoshan, Dehong, Lincang, and Pu’er. For the analysis of the H1 element, data related to nine types of natural disasters were collected and analyzed for these five cities using Equations (1)–(10). The assessment indicator representing the degree of natural disaster losses in the study area is denoted as X. If X is greater than the mean X across cities in Yunnan Province, it indicates the need to establish provincial-level, municipal-level, and county-level disaster relief supplies reserve depots. The five cities in the study area include a total of 30 counties. For the analysis of the E1 element, Equations (1)–(9) were applied to evaluate these counties. Counties ranked within the top 15% of E1 indices were excluded from consideration as potential provincial-level reserve depot locations. Similarly, the counties with the highest indices in the five cities were excluded from municipal-level reserve depot site selection based on Equations (10),(11). For the V1 element, counties with the lowest indices in the five cities were excluded from consideration for provincial-level and municipal-level reserve depot locations based on Equations (7),(10),(12). For the C1 element, counties with the lowest indices in the five cities were also excluded from consideration for provincial-level and municipal-level reserve depot locations based on Equations (7),(10),(12).

**Fig 3:**
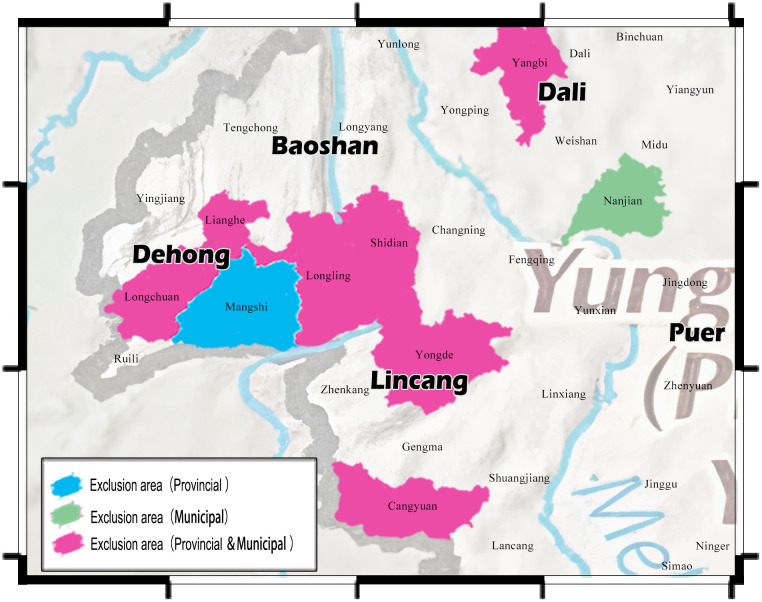
Areas that cannot be selected as provincial or municipal warehouses. (The original base map is from httpswww.cia.govstatic).

Mang City in Dehong City is identified as unsuitable for a provincial-level reserve depot location, and Nanjian County in Dali City is identified as unsuitable for a municipal-level reserve depot location by solving L1. In addition, 10 counties, including Longchuan County and Lianghe County in Dehong City, Yangbi County and Changning County in Baoshan City, Ximeng County and Jiangcheng County in Pu’er City, Cangyuan County and Yongde County in Lincang City, and Yangbi County and Heqing County in Dali City, are identified as unsuitable for provincial-level and municipal-level reserve depot locations (Fig 3).

### 3.2. Technical methods application for L2

#### 3.2.1. Technical methods application for H2.

**Fig 4:**
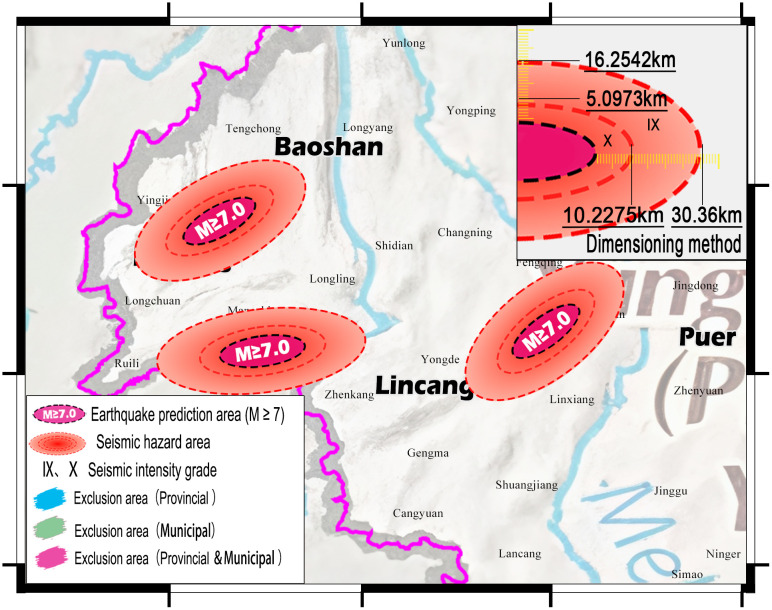
Prediction of earthquake prone areas. (The original base map is from httpswww.cia.govstatic).

[27] conducted a study in the western Yunnan region, in which historical data were collected, and relevant research findings were integrated to support the regional assessment. The study predicted the occurrence of earthquakes with a magnitude of 7 or higher, with the intensity level reaching *X* and the maximum magnitude approaching 8. This estimated earthquake severity is substantially higher than that of the Yunnan Tonghai earthquake, which had a magnitude of 7.7 in 1970 [30], as well as the dual-seismic Yunnan Lancang/Gengma earthquake, which had a magnitude of 7.2 in 1988. The assigned severity level reflects extreme modeling scenarios and represents an upper-bound estimation under worst-case conditions. In addition, based on historical research data and seismic intensity attenuation models, [27] identified multiple locations within the study area that were susceptible to earthquakes with a magnitude of 7 or higher and an intensity level of VIII or higher. These locations were classified as earthquake hazard areas (Fig 4). The detailed methodological procedures are provided in [27]. Because of the high predicted earthquake intensity, these earthquake hazard areas are unsuitable for selection as provincial-level or municipal-level reserve depot sites.

#### 3.2.2. Technical methods application for E2.

In the context of disaster relief supplies reserve depot site selection under earthquake scenarios of magnitude 7 or higher, exposure analysis primarily focuses on identifying areas where earthquakes can cause severe structural damage and estimating the number of people potentially affected. This assessment ensures that adequate disaster relief supplies can be deployed efficiently to impacted areas following earthquake events. In this study, remote sensing satellite data were utilized to obtain spatial information on building distribution, while nighttime light data were utilized to represent population activity and settlement intensity. These datasets, combined with the earthquake hazard zones (Fig 4), were integrated to evaluate the extent of affected areas and the population at risk.

**Fig 5:**
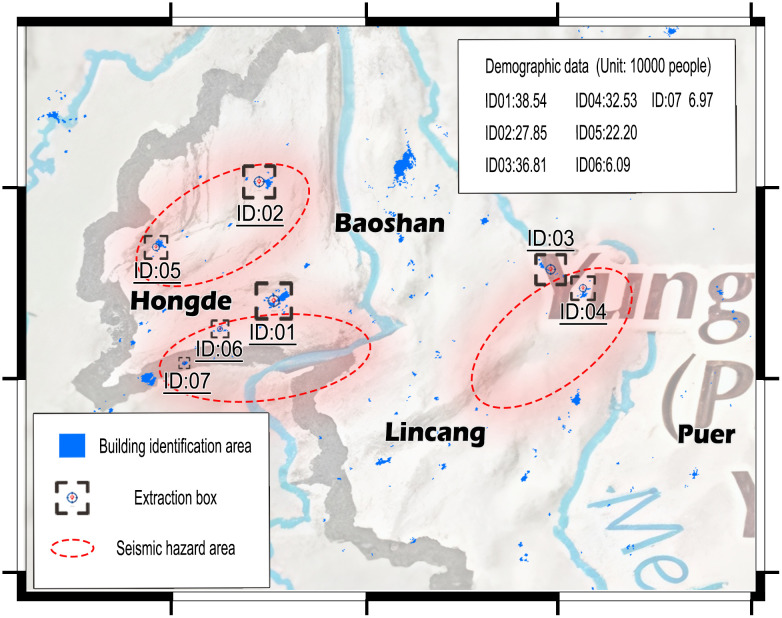
Earthquake hazard areas and population distribution map. (The original base map is from httpswww.cia.govstatic).

The building area data used in this study were extracted from the MODIS Level 3 land cover type product MCD12Q1 using the IGBP global vegetation classification system. Building area extraction primarily focused on urban and built-up land cover (classification code 13). The results identified building-prone areas labeled as ID01 to ID07 (Fig 5). These identified hazard areas are not appropriate locations for provincial-level reserve depots. Because the smallest statistical unit in the population census data is the township level, the spatial resolution of these data does not satisfy the accuracy requirements for disaster relief supplies reserve depot site selection. Therefore, establishing a regression relationship between census statistics and geographic spatial variables can provide more reliable population distribution estimates [31]. Considering the geographic characteristics of the study area and the operational requirements of post-disaster emergency response, this study conducted spatial analysis using 2019 satellite nighttime light data, land use data, river and road network data, and population statistical data. Using the Analytic Hierarchy Process (AHP), the study generated a 2019 population distribution grid dataset for the research area with a spatial resolution of 100 m. Fig 5 indicates that the dataset was compared to global population spatial distribution data from the WorldPop dataset (https://hub.worldpop.org/). The derived population distribution data demonstrated high correlation with the WorldPop dataset and showed improved suitability for this study.

Based on the population distribution results derived from remote sensing nighttime light data, the seven affected areas contained relatively large populations. A magnitude 7 earthquake occurring in these areas can lead to widespread disaster impacts and substantial resource demands [32]. If the regional disaster relief supplies reserve depot becomes nonfunctional following an earthquake, the affected areas will require direct support from a larger-scale regional disaster relief supplies reserve depot.

#### 3.2.3. Analysis of V2 and C2.

Road transportation systems are often severely damaged following earthquakes, and this disruption becomes particularly significant under earthquakes with a magnitude of 7 or higher. Research based on historical seismic activity and aftershock records within the study area indicates that after an earthquake of magnitude 7 or higher, the maximum aftershock magnitude can be estimated as Ms_max_=Ms −0.8 [33], and the largest aftershock is highly likely to occur within 3 days. When the risk of secondary seismic disasters is also considered, reliance on road-based relief supply transportation introduces substantial operational hazards. In severe scenarios, ground transportation can become infeasible following a magnitude 7 or higher earthquake within the research area. Therefore, post-disaster vulnerability analysis indicates that helicopters represent an effective approach for transporting post-earthquake relief supplies, particularly when timeliness and extreme operational conditions are incorporated into the modeling process.

Field investigation findings from the Southern Theater Command of the People’s Liberation Army, the Yunnan Military District, and the fire-fighting and public security sectors in Yunnan Province indicated that the primary helicopter fleet capable of supporting large-scale emergency transport tasks during the next 5–10 years is the 13-ton-class Mi-171 model. During search and rescue operations, helicopter missions are associated with a higher probability of human error than other operational scenarios [34]. Reducing the frequency of take-off and landing operations during rescue missions can effectively decrease the likelihood of operational errors during helicopter deployment [35]. Based on the full-fuel standard flight data of the 13-ton-class Mi-171 model and the “Flight Operating Instructions for Mi-171 Helicopter Series” [36], the safe flight formula is derived as follows:


$$\begin{array}{c} D_{max}={\left(TSR-FLR\right)}_{STS\&MP}/\text{2} \end{array}$$ (23)

$$\begin{array}{c} T_{min}=D_{min}÷STS\text{km}/\text{h} \end{array}$$ (24)

where *D*_*max*_ is the largest safe flight range; *TSR* denotes the total standard range; *FLR* is the fuel limit range; *STS* is the standard test speed; *MP* is the maximum payload; *T*_*min*_ is the shortest transport time; *D*_*min*_ is the smallest safe flight range.

The calculations indicate that the standard Mi-171 model can safely fly up to 225 km during disaster relief supplies transport missions. The minimum estimated transport time is 0.94 hours. Therefore, the emergency response time and helicopter dispatch time for the provincial-level reserve depot were set to 2 hours after the earthquake. Under favorable weather conditions, the first batch of disaster relief supplies can feasibly reach the affected areas within 3 hours. This time frame satisfies the modeling requirement that the first batch of critical supplies from the provincial-level disaster relief supplies reserve depot should arrive within 5 hours.

### 3.3. Technical methods application for L4

#### 3.3.1. Identifying site selection candidates.

**Fig 6:**
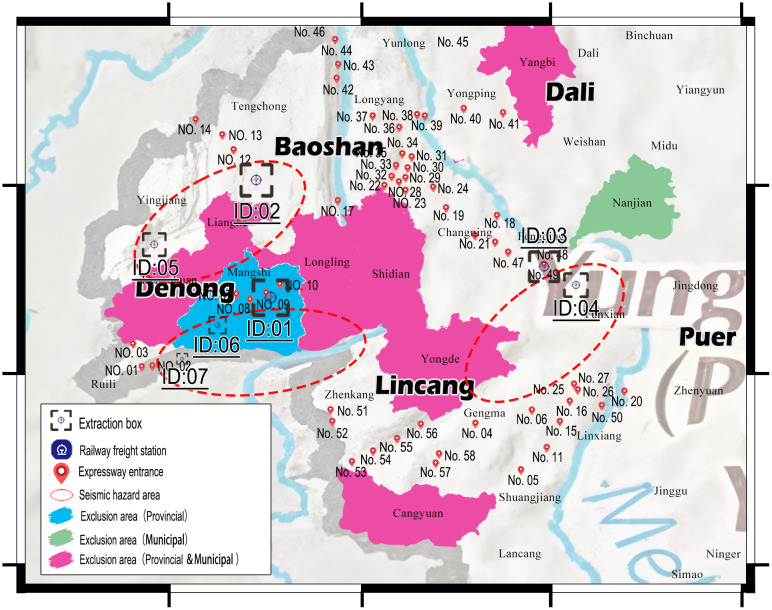
Distribution of candidate areas. (The original base map is from httpswww.cia.govstatic).

Based on the functional requirements of the disaster relief supplies reserve depot, as shown in Table 5, this study selected areas within a 5 km radius of each highway entrance and railway freight station as candidate sites to ensure convenient transportation access for disaster relief supplies. This criterion was adopted based on accessibility considerations in the modeling framework. Geographic information on the locations of highway entrances, railway freight stations, and airports in Yunnan Province was collected as of August 2019. After applying the regional constraints derived from L1, the hazard area constraints from E2, and the safe flight distance constraints for reaching the affected areas from C2, a total of 59 candidate areas meeting the requirements were identified (No. 1–59, as shown in Fig 6) for municipal-level disaster relief supplies reserve depot site selection, and 55 areas were identified as provincial-level candidates. The calculation formula is as follows:

The formula is the same as 2.3.6 Formula (13)−(19)

Constraint condition:


$$\begin{array}{c} \sqrt{{(x_{i}-x_{j})}^{\text{2}}{+(y_{i}-y_{j})}^{\text{2}}}\leq \text{225000m},i=\text{1},\text{2},...,\text{7} \end{array}$$ (25)

where ($x_{i}$, $y_{i}$) is the coordinates of a disaster-affected area; ($x_{j}$, $y_{j}$) is the coordinates of a candidate site.

#### 3.3.2. Areas meeting the terrain index standard.

**Fig 7:**
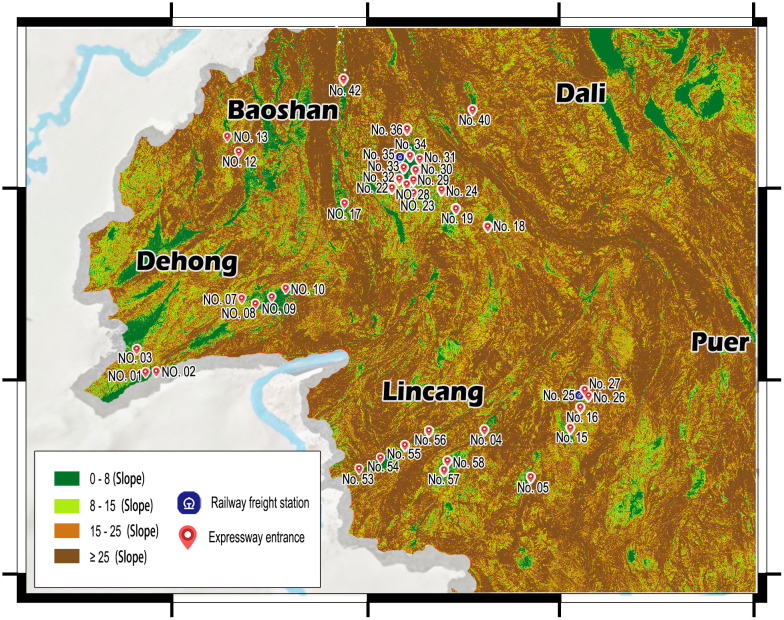
Distribution of candidate areas of terrain index reaching standard. (The original base map is from httpswww.cia.govstatic).

Based on the disaster relief supplies reserve depot functions presented in Table 5, this study applied the criterion that the slope of each unit within the candidate area, evaluated using a 150 m × 150 m window, should be less than 25%. This threshold was adopted to ensure adequate construction suitability for provincial-level and municipal-level disaster relief supplies reserve depots and to support safe helicopter take-off and landing operations. This criterion was established based on both functional feasibility and operational safety considerations embedded in the modeling framework. The digital elevation model (DEM) was used as the quantitative basis for terrain index selection. The DEM dataset used in this study has a spatial resolution of 30 m, representing elevation values within a 30 × 30 m grid. Three terrain indices were considered, including regional slope, regional mean elevation, and regional standard deviation. Based on the above prerequisites and using Equations (19)−(21), 20 candidate areas were excluded, resulting in 39 remaining candidate areas (Fig 7). Among these, 35 areas were identified as provincial-level candidates, while 39 areas were identified as municipal-level candidates.

#### 3.3.3. Ranking of transportation convenience functionality.

Based on the functional requirements of disaster relief supplies reserve depots outlined in Table 5, these depots must support convenient transportation access and efficient mobilization under emergency conditions. This study considers nearby road transportation hubs, railway freight stations (located near highway exits), and airports as key conditions supporting transportation convenience and mobilization efficiency. The distance between each candidate area and these three types of target points was calculated. Because earthquakes with a magnitude of 7 or higher were treated as constraining conditions, transportation hubs were required to include the intersection of two highways and at least two directions identified as non-seismic hazard areas. Candidate areas that failed to meet this condition were excluded (No. 01–03). Then, the road transportation distances from the remaining candidate areas to the three types of target points were recalculated, and preference rankings were determined using the P-median calculation method (Table 7). The calculation formula is as follows:

**Table 7:** Data of the top 10 candidate districts of the preferred conditions (unit: m).

candidate site	ID1	ID2	ID3	ID4	ID5	ID6	ID7	Airport	station (expressway exit)	transportation hub	P-median	Difference
No. 32	106.009	73644.15	97608.40	123178.08	134038.39	136476.36	162628.94	974.26	14093.17	7001.35	22068.78	–
No. 33	112795.33	76592.25	99798.09	125487.23	140519.41	140809.59	169114.52	8623.71	8798.43	13131.81	30553.95	8485.17
No. 28	106395.84	77678.48	92430.11	117993.55	134929.17	139573.56	163484.68	6792.80	12368.40	19536.21	38697.41	8143.46
No. 35	111633.52	73910.78	99245.62	99245.62	136180.11	137781.76	165103.47	14433.10	7801.23	19701.43	41935.76	3238.3
No. 34	81334.55	146609.78	176718.85	148129.03	120621.31	101775.92	127480.81	16623.56	8468.87	21127.98	46220.41	4284.65
No. 30	115771.36	82837.81	93730.15	119439.58	144060.91	146451.41	172640.77	15091.12	12692.53	19427.06	47210.71	990.30
No. 29	110684.24	81196.65	91116.75	116770.70	139264.82	143609.51	167816.75	12326.96	19736.91	17408.36	49472.23	2261.5
No. 23	105051.85	81278.50	86633.30	112121.31	134127.07	141655.02	162592.90	16354.21	30700.16	15988.22	63042.59	12074.5
No. 22	91392.86	63873.08	101035.15	126045.44	119414.69	123974.65	148002.22	20622.69	34426.31	14437.69	69486.69	6444.10
No. 31	85988.02	150738.86	178979.74	150386.23	122388.70	96705.91	122393.98	23245.32	20551.56	25776.59	69573.47	86.78

**Note: Code H refers to a highway entrance. Code T refers to the station. ID1–7 refers to the candidate site.**

The formula is the same as 2.3.6 Formula (13)−(19)


$$\begin{array}{c} d=\sum_{q=\text{2}}^{n}\sqrt{{(x_{q}-x_{q-\text{1}})}^{\text{2}}{+(y_{q}-y_{q-\text{1}})}^{\text{2}}} \end{array}$$ (26)

$$\begin{array}{c} \text{Z}=\text{min}\sum_{j=J}\sum_{f=F}\sum_{g=G}\sum_{p=P}{(\text{h}}_{j,f}d_{j,f}+{\text{h}}_{j,g}d_{j,g}+{\text{h}}_{j,p}d_{j,p}) \end{array}$$ (27)

Decision-making variable:


$$\begin{array}{c} {\text{h}}_{j,f}=\left\{ \begin{array}{c} \text{1}\text{ Transportation hub }f\text{ provision of services to candidate area }j\\ \text{0}\text{ Transportation hub }f\text{ not provision of services to candidate area }j \end{array}\right. \end{array}$$ (28)

$$\begin{array}{c} {\text{h}}_{j,g}=\left\{ \begin{array}{c} \text{1}\text{ Airport }g\text{ provision of services to candidate area }j\\ \text{0}\text{ Airport }g\text{not provision of services to candidate area }j \end{array}\right. \end{array}$$ (29)

$$\begin{array}{c} {\text{h}}_{j,p}=\left\{ \begin{array}{c} \text{1}\text{ Station }(\text{Expressway exit})p\text{ provision of services to candidate area }j\\ \text{0}\text{ Station }(\text{Expressway exit})p\text{ not provision of services to candidate area }j \end{array}\right. \end{array}$$ (30)

Constraint conditions:


$$\begin{array}{c} \sum_{f\in F}{\text{h}}_{j,f}=\text{1},j\in J \end{array}$$ (31)

$$\begin{array}{c} \sum_{g\in G}{\text{h}}_{j,g}=\text{1},j\in J \end{array}$$ (32)

$$\begin{array}{c} \sum_{p\in P}{\text{h}}_{j,p}=\text{1},j\in J \end{array}$$ (33)

where

d: sum of distances between various geographical information points of the road network

n: total number of geographical information points of the road network

*Z:* objective function, i.e., the sum of shortest distances

*q:* road network information points

*j:* candidate site, *j* ∈ *J*

*J:* set of candidate sites

${\text{h}}_{j,f}$, ${\text{h}}_{j,g}$, ${\text{h}}_{j,p}$*:* binary decision variables

*f:* transportation hub, *f* ∈ *F*

*F:* set of transportation hubs

*g:* airport, *g* ∈ *G*

*G:* set of airports

*p:* station (expressway exit), *g* ∈ *G*

*P:* set of stations (expressway exits)

$d_{j,f}$, $d_{j,g}$, $d_{j,p}$: distance from each selected area to the candidate area

*i:* candidate site, *j* ∈ *I*

*I:* set of candidate sites

$d_{i,k}$: distance between a candidate site and a disaster-affected area

*p*: number of candidate sites

*k:* traffic hub, airport, or railway freight station (highway entrance), *k* ∈ *K*

*K*: set of traffic hubs, airports, and railway freight stations (highway entrances), k ∈ K

#### 3.3.4. The selection and layout of candidate areas.

**Fig 8:**
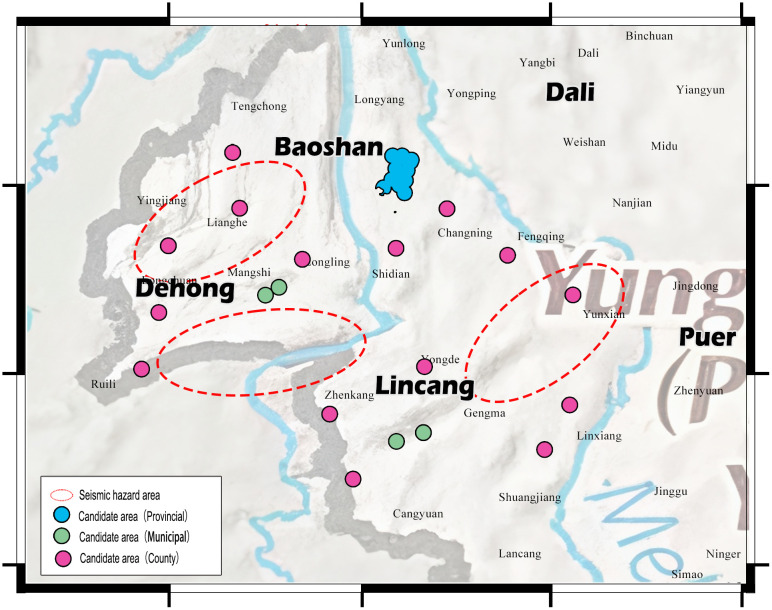
Layout of candidate areas for disaster relief supplies reserve depots. (The original base map is from httpswww.cia.govstatic).

Since this study selected the western region of Yunnan Province, China (23°N–26°N, 97°E–101°E), as the research area, three complete cities are included within the study area. Therefore, the model presents only the spatial layouts of these three cities, namely Dehong, Baoshan, and Lincang, at the provincial, municipal, and county levels for the site selection of disaster relief supplies reserve depots. When earthquakes with magnitudes of 7 or higher are applied as constraints for the site selection of disaster relief supplies reserve depots, county-level depots can serve as intermediate material transportation stations during the later stage of disaster response. Therefore, these depots still need to provide convenient transportation and support rapid mobilization [37]. Two conditions must be followed in the site selection of county-level disaster relief supplies reserve depots. First, the site selection requirements are the same as those for municipal-level depots, although county-level depots are not required to meet the terrain index requirements. Second, if the research area is located within a hazard area, the candidate area farthest from the earthquake epicenter should be selected. This study defines the candidate areas for the provincial-level depot as the top 10 ranked locations within the research area. In contrast, the candidate areas for the municipal-level depots are defined as the top 2 ranked locations within each of the three cities, whereas the candidate areas for the county-level depots are defined as the top 1 ranked location within each county. Based on the ranking for convenient transportation, the top 10 candidate areas meeting the requirements for the provincial-level depot are concentrated in Longyang District, Baoshan City (Table 7). The highest-ranked candidate areas for the municipal-level depot in Dehong City are No. 08–10, which are located in Mang City. The highest-ranked candidate areas for the municipal-level depot in Lincang City are No. 53–54, which are located in Gengma County. Considering the two conditions required for selecting county-level depots, the site selection results for the county-level depots are presented in Fig 8.

### 3.4. References to municipal planning and other conditions

**Fig 9:**
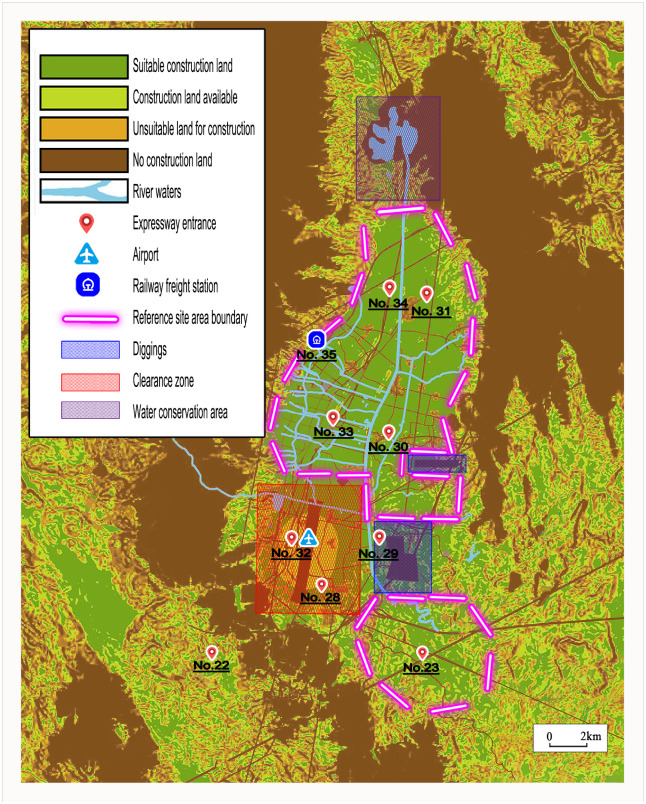
Candidate area.

Within the model constructed in this study, L4 serves as the reference criterion for selecting candidate areas. Using the provincial-level disaster relief supplies reserve depot as an example, the selection of candidate areas for the municipal-level and county-level depots is not further discussed. Based on the slope data derived from the digital elevation model (DEM) of Baoshan City and the requirements outlined in the “Vertical Planning Norms for Urban Land Use” [38], factors constraining the construction of disaster relief supplies depots, including airport clearance restrictions, hydrological conditions, the distribution of mineral resources, the distribution of important pipelines, and helicopter take-off and landing requirements, are integrated to derive the regional land-use situation. The final candidate area for the disaster relief supplies reserve depot is determined by combining the spatial positions of the top 10 ranked candidate areas (Fig 9). This result is consistent with the site selection outcomes for large-scale depots in the region reported by [27]. Based on these findings, the study concludes that after candidate areas for disaster relief supplies reserve depots are identified, the final site should be located away from hazardous areas, such as fire sources and facilities associated with flammable, combustible, or explosive materials, including factories and warehouses.

## 4. Discussion

### 4.1. Empirical validation and application

Among the emergency supplies reserve depots identified in this study, including the provincial-level depot in Baoshan and the city-level depots in Dehong and Lincang, the locations of the Baoshan provincial depot and the Dehong city depot are entirely consistent with the currently constructed sites. However, the Lincang city depot is located near candidate area No. 15, indicating a slight deviation from the results of this study. Since 2020, multiple emergency drills for earthquakes with magnitudes of 5 or higher have been conducted in the study area, and all drills have produced satisfactory outcomes, demonstrating the rationality of the site layout within the region. In the future, if further optimization of the layout is required, a westward adjustment of the Lincang city depot can be considered to enhance post-earthquake disaster relief efficiency.

### 4.2. Comparative analysis with other studies

**Fig 10:**
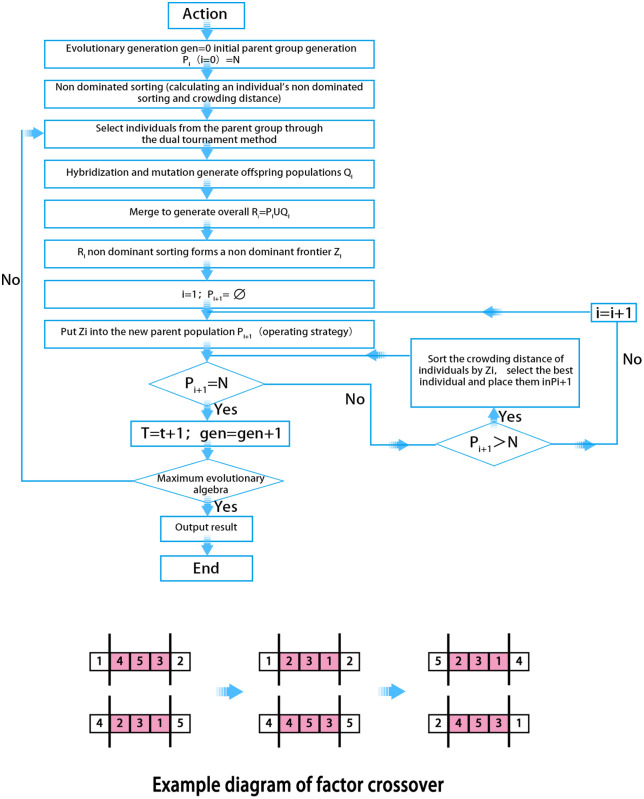
Guan et al.’s NSGA-II algorithm flowchart and factor crossover example.

In the practical site selection, validating the effectiveness of regional site selection is a complex task that requires extensive empirical verification. Because this study focuses on extreme disasters, and the probability of such events is inherently low, practical validation requires long-term data accumulation. Therefore, this study selected the disaster relief supplies reserve depot site selection research by [7] for comparative analysis to examine the applicability of the proposed model. The study area was adjusted to match the empirical case study area adopted in this research, and the results were then compared to the empirical results of this study. Guan et al.’s model addresses the hierarchical location problem and applies the genetic algorithm NSGA-II (Fig 10) to solve the site selection problem for disaster relief supplies reserve depots within a region. This model is based on earthquake disasters and utilizes a hierarchical location structure solved through genetic algorithms, which aligns closely with the research objectives of this study. Therefore, a comparative analysis was conducted between the proposed model and the model of [7] to identify their relative advantages and disadvantages in terms of applicability.

**Fig 11:**
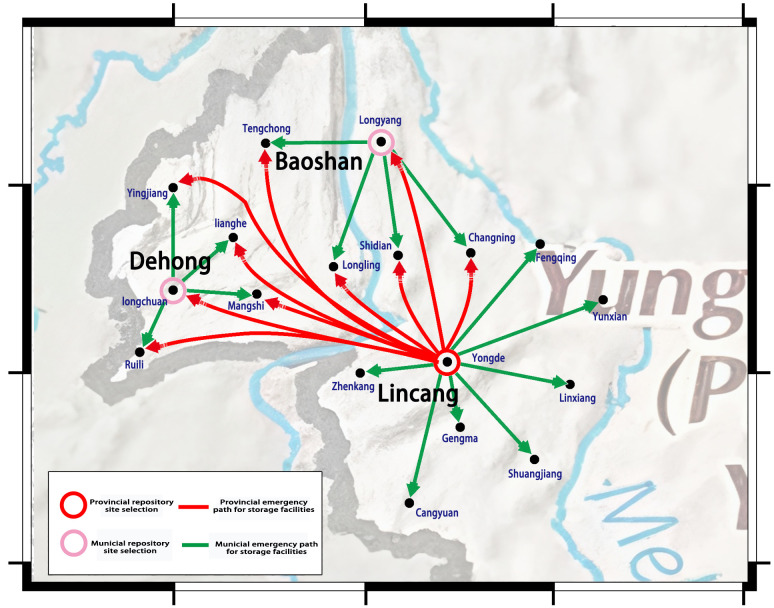
Dependency between warehouses and potential earthquake sites. (The original base map is from httpswww.cia.govstatic).

Based on the regional demand for disaster relief supply reserve levels, geographic information was first extracted for the three cities in western Yunnan (Dehong, Baoshan, and Lincang), following the site selection method and design of [7]. The site selection result for the objective deviation minimization model was fixed at three sites, under the constraint that one county-level unit per city be selected as a municipal-level disaster relief supplies reserve depot site (Table 8). The three selected sites were Longyang District (Baoshan City), Longchuan County (Dehong City), and Yongde County (Lincang City). These locations represent the municipal-level depots identified by the model. The relationships between the selected depots and potential earthquake sites are illustrated by the green arrows in Fig 11.

**Table 8:** Site selection results of the objective deviation minimization model for each city (P = 3).

Selected Site	Covered Potential Disaster Sites	Farthest Covered Location	Coverage Quality (%)	Maximum Travel Time on Highway (h)
Longyang	Longyang, Longling, Shidian, Changning, Tengchong	Longling	85.36%	1.37h
Longchuan	Longchuan, Ruili, Mangshi, Lianghe, Yingjiang	Yingjiang	86.47%	1.17h
Yongde	Yongde, Zhenkang, Fengqing, Yunxian, Linxiang, Shuangjiang, Gengma, Cangyuan, Shuangjiang	Yunxian	75.21%	4.29h

Accordingly, the model was adjusted in stages to focus on the same factors as this study. In the first stage, consistent with the conceptual design of disaster relief supplies reserve depots in the western Yunnan region, one provincial-level disaster relief supplies reserve depot was selected within the region, and potential disaster sites were classified into two levels. This depot was required to be selected from the previously determined municipal-level depot sites. Following the constraints and objective functions proposed by Guan et al., the model was solved using the NSGA-II method (Fig 10). The result identified Yongde County, Lincang City, as the provincial-level disaster relief supplies reserve depot (Table 9).

**Table 9:** Site selection results of the objective deviation minimization model for the region (P = 1).

Selected Site	Level 1 Potential Disaster Sites	Level 2 Covered Potential Disaster Sites	Furthest Covered Location (L1/L2)	Coverage Quality (%)	Highway Travel Time (h) (L1/L2)
Yongde	Longchuan, Longyang	Longling, Shidian, Changning, Tengchong, Ruili, Mangshi, Lianghe, Yingjiang, Zhenkang, Fengqing, Yunxian, Linxiang, Shuangjiang, Gengma, Cangyuan, Shuangjiang	Longchuan / Ruili	79.11%	5.31 / 5.22h

The site selection model proposed in this study presents three distinct advantages compared to the application of Guan et al.’s adjusted model in the western Yunnan region [7]. First, Guan et al.’s site selection model lacks specific disaster-stricken points [7]. All county-level areas within the region are treated as potential disaster sites, and the occurrence of disasters lacks a clear pattern. It even posits the hypothesis of simultaneous disasters in Ruili (in the western region) and Yunxian (in the eastern region), a scenario that does not conform to the spatial patterns of earthquake occurrences. Second, the latter focuses only on road transportation. Following a strong earthquake, it is difficult to ensure the accessibility of the transportation network for large vehicles transporting livelihood supplies. Therefore, the design of that model is difficult to apply in practice. Third, the Guan et al.’s model cannot prioritize municipal conditions and urban planning during the site selection process, making it impossible to determine whether the final selection results are compatible with urban planning [7]. In practical site selection applications, beyond the three points mentioned above, strong earthquakes often trigger potentially large-scale aftershocks, causing a large number of victims. At the same time, local disaster relief supplies reserve depots in the earthquake zone will likely be damaged or paralyzed. Therefore, the delivery of livelihood supplies relies on air transportation from large disaster relief supply reserve depots outside the region. Hence, the site selection work for disaster relief supplies reserve depots conducted in this study demonstrates superior applicability in practice.

### 4.3. Contribution and innovation

Based on the disaster relief supplies reserve depot site selection model constructed using the mentioned natural disaster risk factors, this paper verified its applicability to extreme disasters such as earthquakes of magnitude 7 or higher, ultimately obtaining the layout for the region’s three-tier disaster relief supplies reserve depots. In comparison to studies focusing solely on the transportation of disaster relief supplies [39], single natural disaster risk considerations [11,27], the perception of victims’ pain [6], and the minimization of warehouse numbers in relation to rescue satisfaction [9], this research emphasizes both the natural disaster risk elements that disaster relief supplies reserve depots need to address and their functionality, demonstrating strong practical and innovative capabilities.

First, this study innovatively integrates the intrinsic concept of disaster risk into a site selection model based on four types of disaster risk elements, establishing an indicator system that links risk factors and site selection decisions. Through the comprehensive analysis of hazard and exposure elements, the service targets for the site selection of disaster relief supplies reserve depots are obtained, and the disaster-stricken areas within the region are accurately identified. Through a comprehensive analysis of vulnerability and capacity elements, the functional conditions and regional constraints for the depots are derived. The proposed model balances regional disaster risk with the disaster avoidance capability of the disaster relief supplies reserve depots, providing a theoretical innovation in the application of disaster risk analysis methods to site selection.

Second, the study fully considers the regional and functional requirements of disaster relief supplies reserve depots under extreme disaster scenarios by integrating natural disaster risk elements with site selection standards, identifying optimal locations that meet established site selection standards. Compared to existing research that primarily focuses on transportation costs and coverage areas, this study enables site selection of optimal locations for disaster relief supplies reserve depots at a finer scale and demonstrates strong practical application value.

### 4.4. Limitation analysis

Applying the model calls for turning a number of indicators into measurable values. Two of the choices involved were made on practical grounds rather than by following established numerical thresholds. The first was treating a 5‑km buffer around highway entrances as a candidate zone, which is meant to ensure reasonable transport links. The second was using a 150 × 150 m cell as the basic spatial unit, a size intended to accommodate safe helicopter take‑offs and landings. For both parameters, there are no national or international standards to lean on, and the research literature does not provide clear empirical backing either. This is an unavoidable data limitation of the study.

To make sure the indicators still captured conditions on the ground, members of the research team spoke directly with emergency responders who work in the region. The values were therefore set based on local know‑how and the way rescue operations actually unfold in the area. As a result, the model reflects what is realistically needed when siting relief supply depots here.

At the same time, it is worth keeping in mind that the parameter settings are tied to this particular context. They work reasonably well for the study area, but there is no guarantee they will be equally suitable somewhere else with different geography or seismic patterns. The underlying assumptions would need to be checked before applying the same numbers to another region. If better historical data or higher‑resolution spatial information becomes available in the future, users are encouraged to adjust the parameters at the relevant levels of the model. Doing so should help the approach travel more reliably across different settings.

## 5. Conclusion

This study investigates the site selection of disaster relief supplies reserve depots and proposes a site selection model that incorporates natural disaster risk. With reference to the “Sendai Framework for Disaster Risk Reduction 2015–2030” and the [16] conceptualization of disaster risk components, the study identifies four key elements, namely, hazard, exposure, vulnerability, and capacity. These elements represent conditions that exist both before and after a disaster, forming the foundation of a disaster relief supplies reserve depot site selection model that integrates pre-disaster regional factors (L1), post-disaster assessment outcomes (L2), disaster relief supplies reserve depot functions (L3), and urban planning factors (L4). At the L1 level, the model applies the entropy weight method and the geometric mean model to evaluate multiple regional indicators. At the L2 level, the model conducts a detailed analysis of post-disaster losses across different disaster-affected entities based on the characteristics of each disaster type. At the L3 level, the model determines the required functions of disaster relief supplies reserve depots by analyzing the “Standards for the Construction of Disaster Relief Supplies Reserve Depots” [25], and it uses regional GIS data and satellite remote sensing data to establish functional requirements for disaster relief supplies reserve depots. These requirements are then utilized to develop the spatial layout of 3-level disaster relief supplies reserve depots through inter-level constraint relationships. At the L4 level, the model incorporates urban planning factors to support the selection of sites for 3-level disaster relief supplies reserve depots. Field visits and validation indicate that the model-generated layout closely matches the actual placement of provincial-level depots (near candidate area No. 33). Although minor deviations are observed in the layouts of municipal-level and county-level depots, these depots retain the fundamental conditions necessary for improvement. The results demonstrate that the proposed model effectively supports future enhancements to the emergency disaster relief supplies reserve system and provides an effective approach for disaster relief supplies reserve depot site selection. Globally, site selection challenges related to natural disaster risk remain widespread. Decision-makers can obtain high-quality site selection solutions within an acceptable time frame by aligning algorithms with practical implementation strategies and incorporating regional context and disaster relief principles. In addition, the analytical framework enables the inclusion of additional disaster types in emergency material reserve depot site selection, further strengthening the model’s practical applicability.

## Supporting information

S1 File Entrance of expressways in three cities in western Yunnan (https://doi.org/10.6084/m9.figshare.31553032).
(RAR)

S2 File Yunnan Provincial Yearbook (https://doi.org/10.6084/m9.figshare.31552957).
(RAR)
